# In response: “Unexpected deposits in the anesthetic circuit: a possible cause of PEEP/Pmax valve malfunction” from T. Ikeda et al. https://doi.org/10.1007/s10877-020-00562-3

**DOI:** 10.1007/s10877-020-00577-w

**Published:** 2020-08-27

**Authors:** Hans Ulrich Schüler

**Affiliations:** Drägerwerk AG & Co. KGaA, Lübeck, Germany

We would like to thank the editor of this journal for giving us as the manufacturer the opportunity to respond to this presented case of unexpected deposits in the anaesthetic circuit, which caused PEEP/Pmax valve malfunction.

In this article, the authors describe cases where deposits of particles have been found inside the PEEP/Pmax valve of seven out of twenty two Fabius Anesthesia Workstations.

The hospital has sent to us affected PEEP/Pmax valves for further investigation. Upon investigation we found deposits of various materials, incl. silicone and calcium, which were not coming from the anesthesia workstation itself, inside the PEEP/Pmax valve.

We were not able to identify the real source of these specific particles. We have found in one other investigation similar particles. In this case dust releasing soda lime has been used over a longer period of time, and at the same time regular cleaning cycles of the breathing system did not take place.

Therefore, the general recommendation from us as the manufacturer is to pay attention to the chapter cleaning of the respective instructions for use: regular cleaning according to the manufacturers recommendation in the instruction for use avoids building up of particles inside the breathing system. Furthermore, using high quality, non-dust releasing absorbent, especially for longer anesthesia cases, helps reducing the built up of dust, and therefore avoids the potential situation of building up particles inside the breathing system.

We would like to thank the authors of this article, bringing this to the attention of the readers of this journal. It supports the need for following the instructions for use, especially the chapter cleaning, disinfection, and sterilization.

Hans Ulrich Schüler

